# Intersection of the Web-Based Vaping Narrative With COVID-19: Topic Modeling Study

**DOI:** 10.2196/21743

**Published:** 2020-10-30

**Authors:** Kamila Janmohamed, Abdul-Nasah Soale, Laura Forastiere, Weiming Tang, Yongjie Sha, Jakob Demant, Edoardo Airoldi, Navin Kumar

**Affiliations:** 1 Human Nature Lab Department of Sociology Yale University New Haven, CT United States; 2 Department of Statistical Science Fox School of Business Temple University Philadelphia, PA United States; 3 Department of Biostatistics Yale School of Public Health New Haven, CT United States; 4 UNC Project-China University of North Carolina at Chapel Hill Guangzhou China; 5 Southern Medical University Dermatology Hospital Guangzhou China; 6 School of Medicine University of North Carolina at Chapel Hill Chapel Hill, NC United States; 7 University of Copenhagen Copenhagen Denmark

**Keywords:** vaping, COVID-19, topic modeling, web-based narrative, modeling, trend, narrative, social media, internet, web-based health information

## Abstract

**Background:**

The COVID-19 outbreak was designated a global pandemic on March 11, 2020. The relationship between vaping and contracting COVID-19 is unclear, and information on the internet is conflicting. There is some scientific evidence that vaping cannabidiol (CBD), an active ingredient in cannabis that is obtained from the hemp plant, or other substances is associated with more severe manifestations of COVID-19. However, there is also inaccurate information that vaping can aid COVID-19 treatment, as well as expert opinion that CBD, possibly administered through vaping, can mitigate COVID-19 symptoms. Thus, it is necessary to study the spread of inaccurate information to better understand how to promote scientific knowledge and curb inaccurate information, which is critical to the health of vapers. Inaccurate information about vaping and COVID-19 may affect COVID-19 treatment outcomes.

**Objective:**

Using structural topic modeling, we aimed to map temporal trends in the web-based vaping narrative (a large data set comprising web-based vaping chatter from several sources) to indicate how the narrative changed from before to during the COVID-19 pandemic.

**Methods:**

We obtained data using a textual query that scanned a data pool of approximately 200,000 different domains (4,027,172 documents and 361,100,284 words) such as public internet forums, blogs, and social media, from August 1, 2019, to April 21, 2020. We then used structural topic modeling to understand changes in word prevalence and semantic structures within topics around vaping before and after December 31, 2019, when COVID-19 was reported to the World Health Organization.

**Results:**

Broadly, the web-based vaping narrative can be organized into the following groups or archetypes: harms from vaping; Vaping Regulation; Vaping as Harm Reduction or Treatment; and Vaping Lifestyle. Three archetypes were observed prior to the emergence of COVID-19; however, four archetypes were identified post–COVID-19 (Vaping as Harm Reduction or Treatment was the additional archetype). A topic related to CBD product preference emerged after COVID-19 was first reported, which may be related to the use of CBD by vapers as a COVID-19 treatment.

**Conclusions:**

Our main finding is the emergence of a vape-administered CBD treatment narrative around COVID-19 when comparing the web-based vaping narratives before and during the COVID-19 pandemic. These results are key to understanding how vapers respond to inaccurate information about COVID-19, optimizing treatment of vapers who contract COVID-19, and possibly minimizing instances of inaccurate information. The findings have implications for the management of COVID-19 among vapers and the monitoring of web-based content pertinent to tobacco to develop targeted interventions to manage COVID-19 among vapers.

## Introduction

COVID-19 is spreading rapidly and has been declared a global pandemic [1]. COVID-19 was first reported in Wuhan, China, in December 2019 [2], and it was declared a pandemic by the World Health Organization (WHO) on March 11, 2020 [3]. With the pandemic currently in progress, research on the determinants of disease progression and communities that may be more vulnerable to COVID-19 is of key importance [4].

There is some scientific knowledge that vaping cannabidiol (CBD) or other substances may be associated with more severe manifestations of COVID-19 [5,6]. Use of electronic cigarettes (e-cigarettes) has been associated with a reduction in the ability of the lungs to respond to infection [5,6]; thus, people who use e-cigarettes may be at increased risk of contracting COVID-19 [4,7]. Several studies have indicated that smokers, including vapers, are more vulnerable to COVID-19 infections or more likely to develop serious complications after contracting SARS-CoV-2, the virus that causes COVID-19 [8-10]. Vaping devices may also be possible sites of COVID-19 transmission [9]. However, inaccurate information that vaping can aid COVID-19 treatment is also circulating [9], and experts have expressed the opinion that CBD, which can be administered through vaping, can mitigate COVID-19 symptoms [11,12].

For example, some Twitter posts around vaping indicated that e-cigarette devices may increase lung humidity and prevent COVID-19, and other posts stated that these devices can be used to administer COVID-19 medication to the lungs and possibly destroy the virus [9]. It has also been suggested that CBD products, often delivered through vaping, can be used to treat COVID-19, perhaps by augmenting the immune system [11,12]. Much of the information around vaping and other tobacco products is disseminated through the internet [13,14] and can affect health outcomes [15,16]. For example, vapers who develop COVID-19 may mistakenly believe that vaping CBD or other substances can alleviate COVID-19 symptoms; however, it may instead create additional disease complications. Thus, it is necessary to study the spread of inaccurate information to better understand how to promote scientific knowledge and curb misinformation, which may be critical to vapers’ health [17].

A previous study analyzed Twitter content around COVID-19 and vaping [9]. The indicated study surveilled vaping tweets and detailed conversations around COVID-19 and vaping. The web-based conversations centered on the possibly heightened risk of COVID-19 for vapers and how vaping could potentially protect against COVID-19 [9]. However, past work did not use web-based vaping-related data from a range of sources or detailed data from before the COVID-19 pandemic and through its progression. Detailing a large scope of sources is necessary to document the broad range of web-based vaping conversations, and collecting data from both before and during the COVID-19 pandemic is key to understanding the narrative prior to the emergence of COVID-19 and how it is changing as the pandemic progresses. Our study builds on previous work by using a large data set to represent the web-based vaping narrative (August 1, 2019, to April 21, 2020) that combines analysis of a multitude of sources, such as blogs, forums, and social media posts; also, we used novel computational techniques to examine how the vaping narrative has changed from before to during the COVID-19 pandemic.

Using the novel computational technique of structural topic modeling (STM), we mapped temporal trends in the web-based vaping narrative (a large data set comprising web-based vaping chatter from several sources) to show how discourse differed before versus during the COVID-19 pandemic. Topic modeling is a computer-aided content analysis technique where texts are organized into themes known as topics. These topics are not provided to the machine prior to modeling but emerge inductively as the algorithm learns patterns within the texts. The model assumes a relational theory of meaning by identifying structures of co-occurrence of words in individual texts and across all the texts. The model thus provides content analysis of text data sets that are too large to code by hand. Topic models use machine learning to uncover patterns and relationships that may be omitted by hand coding or traditional content analysis. Unsupervised machine learning methods have performed similarly to human coders on identical documents [18]. Unsupervised machine learning is a variant of machine learning that looks for new patterns in a data set without pre-existing labels and with limited human supervision. We used an approach to topic modeling known as STM [19,20]. STM enables discovery of topics and their prevalence based on document metadata, such as dates, or other important attributes, such as the number of new COVID-19 cases worldwide per day. Adding this metadata is useful, as the data are obtained over several months (August 2019 to April 2020), and the web-based vaping narrative may be susceptible to thematic change based on the progression of the COVID-19 pandemic. STM has been used to address several social scientific research questions around areas such as climate change [21,22] and web-based drug marketplaces [23]. As vapers may be at greater risk for contracting SARS-CoV-2 and COVID-19 disease progression [8-10], and CBD, which is often administered through vaping, may have interactions with COVID-19 treatment outcomes, we hope to provide insight on how vapers are responding to the pandemic. This may help improve the treatment outcomes of vapers who develop COVID-19.

## Methods

### Ethics Statement

Approval and informed consent were not needed because the data were collected using publicly available textual query techniques. All data are publicly available and can be accessed by anyone. The data were provided to the research team with all identifiers removed.

### Data Acquisition and Processing

Data were obtained using a textual query that scanned a data pool of approximately 200,000 different domains, such as public forum posts, blogs, news articles, message boards, health care provider forums, and social media (see Multimedia Appendix 1 for the full list of sources). Textual queries were used to automatically search the indicated sources for text fragments related to keywords such as *vape*, *vaping*, and *e-cigarette*. The data that comprised vaping-related text fragments were collected from August 1, 2019, to April 21, 2020. As the data set represents a multitude of sources for web-based chatter related to vaping, our data set is likely representative of the web-based vaping narrative during the indicated period. The start date for the COVID-19 pandemic was denoted as December 31, 2019, when the Chinese government disclosed the existence of COVID-19 to the World Health Organization (WHO) [3]. Although the date of the first COVID-19 case is prior to December 31, 2019 [2], COVID-19 is unlikely to have influenced vaping-related discourse in the United States prior to December 31, 2019, due to low global awareness. The time period of August 1, 2019, to April 21, 2020, was chosen to provide sufficient data to detail the vaping narrative prior to the COVID-19 pandemic. Given that the date demarcating pre– and post–COVID-19 is December 31, 2019, our time period allowed for approximately four months before and approximately the same period after the first report of COVID-19 to the WHO.

### Word Prevalence and Topic Modeling

To prepare the data for word prevalence and topic modeling analysis, English stop words such as “the,” “a,” and “an” were removed, along with abbreviations, and terms were stemmed using Porter’s stemming algorithm [24]. Stemming converts words with the same “stem” or root (eg, “innovative” and “innovator”) to a single word type (eg, “innovate”). As our study centered on the intersection of vaping and COVID-19, it was expected that words such as “cigarette,” “vape,” and “coronavirus” would dominate our findings. However, these terms may crowd out other words, perhaps causing us to miss key topics occurring in the text. For example, if we were interested in understanding different cooking techniques such as roasting and frying, and we sourced data from web-based forums frequented by amateur chefs, the most frequent words in the data might be “cook” and “recipe.” However, these words might obscure information around the cooking techniques we were interested in. Thus, in some cases, such as our study, it may be necessary to remove frequently occurring words to detail underlying themes in the data. All data were first processed to remove mentions of COVID-19, tobacco, and vaping. These data were used to generate word clouds by word prevalence. As we will later detail, the word clouds generated by word prevalence contained significant mentions of CBD after the emergence of COVID-19. When conducting topic modeling, mentions of CBD may crowd out other words and reduce our ability to identify salient topics. As such, we further processed the data set for topic modeling by removing mentions of cannabis, inclusive of CBD.

We first generated word clouds based on the top 200 terms ranked by prevalence before and after COVID-19 was reported to the WHO. In a word cloud, a larger font size indicates a greater prevalence of a single word. Word clouds thus provide a relative yardstick of the importance of a word in a particular time period. This visualization enabled us to qualitatively assess words by importance. Documents were processed (words removed) for mentions of cannabis, COVID-19, tobacco, and vaping. References to cannabis were determined using these search terms: [*bud* OR *cannabis* OR *cannabidiol* OR *cbd* OR *ganja* OR *hash* OR *hashish* OR *hemp* OR *indica* OR *joint* OR *marijuana* OR *mary jane* OR *ruderalis* OR *pot* OR *sativa* or *weed* OR *THC*]. References to COVID-19 were determined using these search terms: [*COVID-19* OR *covid 19* OR *novel coronavirus* OR *coronavirus* OR *sars cov-2* OR *sars cov 2* OR *sars-cov-2* OR *n-cov* OR *cov* OR *covid*]. References to tobacco were determined using these search terms: [*baccy* OR *bidi* OR *cig* OR *cigar* OR *cigarillo* OR *cigarette* OR *ciggy* OR *fag* OR *hookah* OR *pipe* OR *shag* OR *sheesha* OR *shisha* OR *snuff* OR *snus* OR *tobacco*]. References to vaping were determined using these search terms: [*e-cig* OR *electronic cigarette* OR *vape* OR *vaper* OR *vaping* OR *vapelife* OR *vapist* OR *vapin* OR *vaplyfe*].

We then used topic modeling to understand changes in word prevalence within topics around vaping and COVID-19. Topic modeling is a computer-aided content analysis technique in which texts are organized into themes known as topics [25,26]. In topic modeling, a topic is a distribution over a vocabulary of words that represent semantically interpretable themes [19]. For example, in a topic denoted “vape,” the terms ”smoke” and ”device” are more likely to occur than the words “peanut” and “tomato.” “Smoke” may appear in both “vape” and “cooking” topics with different contextual meanings. Given that the topic is a distribution, “smoke” may appear with other high-probability terms such as “roast” and “fry” in the “cooking” topic but may appear with terms such as “nicotine” and “device” in the “vape” topic. Thus, topics can be understood by considering that a person who was talking about the topic of “cooking” would tend to use some words more frequently than others compared to if they were talking about the topic of “vape.” Topic models are suitable for analyzing large quantities of textual data via an automated technique for providing context.

We used an approach to topic modeling known as STM [18,20]. STM [18,20] enables the generation of topics regarding document metadata such as date and source as well as other covariates relevant to the research question, such as new COVID-19 cases. This is vital to understanding how the narrative and topic proportions change over time. This enabled a robust quantitative analysis of how the COVID-19 pandemic has shaped the web-based narrative on vaping [19]. The key innovation of STM is that it can incorporate metadata or information about each document. This allows metadata covariates, such as new COVID-19 cases per day, to influence topic discovery. Metadata can affect both the prevalence and content of a topic. Metadata covariates for topical prevalence allow the metadata to affect topic frequency. Similarly, covariates in topical content allow the metadata to affect the word rate within a topic or how a topic is discussed [20]. The STM process will output documents and vocabulary for analysis [20]. This output can be investigated in a range of ways, such as detailing words associated with topics or the relationships between metadata and topics. Model output can be used to conduct hypothesis testing around these relationships. STM [18,20] was applied to the whole data set (August 1, 2019, to April 21, 2020); the data prior to the reporting of COVID-19 to the WHO only (August 1 to December 31, 2019); and the data after COVID-19 was reported to the WHO only (January 1 to April 21, 2020). We subsetted the data to see if the vaping narratives were different before and after COVID-19 was reported to the WHO. We used the following metadata covariates for the STM models. For the full data set, the covariates were the binary variable for before and after COVID-19 was reported to the WHO, COVID-19 content covariate (variable to control for COVID-19–related content), date (the first day was denoted as 1, and the days were numbered sequentially after), source (0=social media, 1=news), new COVID-19 cases per day worldwide, and new COVID-19 deaths per day worldwide. For the pre–COVID-19 data set, the covariates were the date and source. For the post–COVID-19 data set, the covariates were the date, source, new COVID-19 cases per day worldwide, and new COVID-19 deaths per day worldwide.

Because STM is an unsupervised approach, the number of topics to estimate (k) is key to the analysis. We first estimated several models ranging from 5 to 200 topics. These models were then evaluated qualitatively by their ability to produce coherent topics and capture topics regarding vaping and COVID-19 [27]. The number of topics was based on our understanding of the data set and how other researchers interpreted STM results [27,28]. The choice of the number of topics was also influenced by postestimation validation outcomes and past work [27]. As per standard content analysis [29], topic model validation also requires qualitative review, where researchers assess the interpretability and relative efficacy of models based on their subject matter expertise and data context. Our final models (k=15 for the whole data set; k=20 for the pre–COVID-19 report data set; k=20 for the post–COVID-19 report data set) provided the greatest external validity and the most semantically coherent output of distinctive topics. When the number of topics was greater than indicated above, there were diminishing returns for solutions, as the substantive meaning and coherence of categories started to break down [21]. When the number of topics was lower, variation decreased and specific topics were placed into more generic categories. Validating a topic model is not the same as evaluating a statistical model regarding a population sample [30]. The goal is to identify the framework that best describes the data, not to estimate population parameters [30].

We conducted qualitative analysis to determine the number of topics based on past social science studies in which topic modeling was used to extract meaning from large text samples. These studies [21,31] determined topics by qualitative coding based on word prevalence and researchers’ topic expertise. We applied similar techniques in this study. Methods such as interrater reliability ratings may guard against subjective bias based on subject matter expertise and data context [32]. Adding interrater reliability to the qualitative component of topic modeling may improve data quality. However, we sought to use topic modeling to answer a specific research question, not to improve on methodological techniques. Thus, we used best practices implemented in previous studies regarding topic interpretation but did not advance these methods.

Topic interpretation was influenced by prior knowledge about why texts were written and what they sought to accomplish. Most of the text was produced and consumed by people who engaged in vaping and other forms of tobacco use, and this lens was used to interpret the presence or absence of topics and words. Most of the topic labels were straightforward and did not require much interpretation. To characterize topics in the vaping narrative, we qualitatively coded each topic by investigating word clouds based on each topic and reviewing exemplar documents which detailed high proportions of each topic [19]. The topic we classified as “tobacco company merger called off” had the following most frequently occurring words: “sue;” “analyst;” “halt;” “1st;” “imperial;” “judge;” “backlash;” “advisor;” “merger;” “stake;” “acquire;” “outbreak;” “carolina;” “confirm;” and “mint.” Exemplar documents that exhibited high proportions of this topic indicated a preoccupation with these words. This detailed a topical preoccupation with a tobacco firm merger being called off. Thus, the interpretation of the topic was clear, given the genre of the narrative and the reliance on research regarding prominent topics around vaping. Two authors assigned the topics, and a third author resolved disagreements when they arose.

Topic validation is key to assessing whether the substantive meaning of the topic and its words are parallel with the qualitative meaning of the text, and we used methodological guidance from past research for this purpose [19,26]. Past work advocated the use of sample documents to validate the substantive meaning of each topic. Determining the number of sample documents to use is based on the amount of resolution needed by a social scientist to answer the research question using topic modeling methods [33]. Thus, determining the number of sample documents is a largely qualitative process that is dependent on the research question at hand. To determine the appropriate number of documents to sample, we searched the social science literature for studies that used topic modeling based on whether they asked similar research questions to our study, addressed similar topic areas to our study, and drew their study data from similar sources to those used in our study. We searched databases such as Web of Science Core Collection, Embase, PsycINFO, MEDLINE, and Sociological Abstracts. We used keywords such as *contrarian*, *polarized*, and *topic modeling*. The 2016 paper by Farrell [21] was determined to be the most similar to our study based on the assessed characteristics. Farrell explored ideological polarization in climate change and used a broad range of sources, such as press releases, published papers, and website articles. Based on the nature of the research question and large range of sources, Farrell determined that a sample of 50 documents was sufficient to validate the substantive meaning of the topic output. Given the similarities between Farrell’s 2016 study and ours in a range of characteristics, we similarly determined that a sample of 50 documents was adequate to validate the topics. We used findThoughts and plotQuote within the STM package to examine the top 50 associated documents for each topic to validate a topic’s substantive meaning. Determination of the top 50 documents was based on ranking topics by the maximum a posteriori estimate of the topic’s theta value, which represents the modal estimate of the proportion of word tokens assigned to the topic with the model. These top 50 documents were read by two of the authors to determine validity. A third author resolved disagreements where necessary. As indicated above, interrater reliability was not determined; however, we believe our methods were sufficiently robust.

Finally, word clouds were again generated, this time to visualize topics with the top 100 words ranked by STM-generated weights per topic, for the models representing the full data set and the data sets from before and after COVID-19 was reported to the WHO. In these word clouds, a larger font size indicated a greater weight, with word clouds indicating the importance of a word within a topic. We then grouped topics and their associated word clouds into larger categories or archetypes based on shared concepts across topics [34]. All analysis was conducted using R with the following packages: dplyr, quanteda, textclean, tm, and stm [20,24,35-37].

## Results

### Data

We collected 4,027,172 documents (361,100,284 words) comprised of text from blogs (86.01%, N=310,546,244), news articles (11.02%, N=39,721,031), forums (3.01%, N=10,833,008), comments (<1%), professional reviews and Facebook posts (both <1%).

### Word Prevalence

The most frequently observed words in the data set were “ban” (216,735/361,100,284 words, 0.06%, rank 1), “product” (135,607/361,100,284 words, 0.04%, rank 2), and “make” (115,413/361,100,284 words, 0.03%, rank 3).

**Figure 1:**
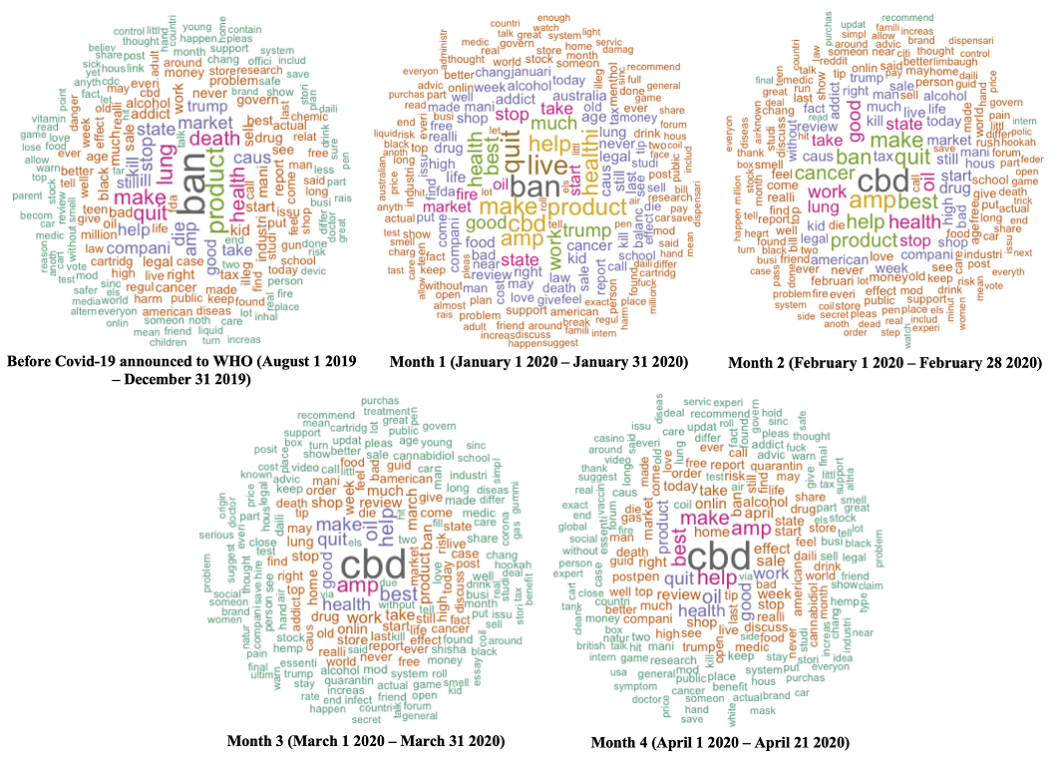
Word clouds showing the 200 most frequently occurring words in the examined documents over time. Month 1 refers to the first month after COVID-19 was reported to the WHO, Month 2 refers to the second month, etc. WHO: World Health Organization.

Figure 1 shows a word cloud displaying the 200 most frequently featured words in the data sets divided by time period. The words are colored, sized, and positioned radially based on frequency of appearance, with larger, more central words appearing most frequently. In the period before COVID-19 was reported to the WHO (prior to December 31, 2019), commonly featured words included “ban,” “lung,” and “quit.” Over the next four months, mentions of “CBD” and “oil” increased, along with positive words such as “good” and “best.” There was a clear shift in word prevalence before COVID-19 was reported to the WHO compared to after the COVID-19 report. Word prevalence shifted from words related to the vaping ban to positive words and words associated with CBD. The vaping ban was a move by the US government on September 11, 2019, to remove all flavored vaping products from the market [38].

### Topic Modeling

**Figure 2:**
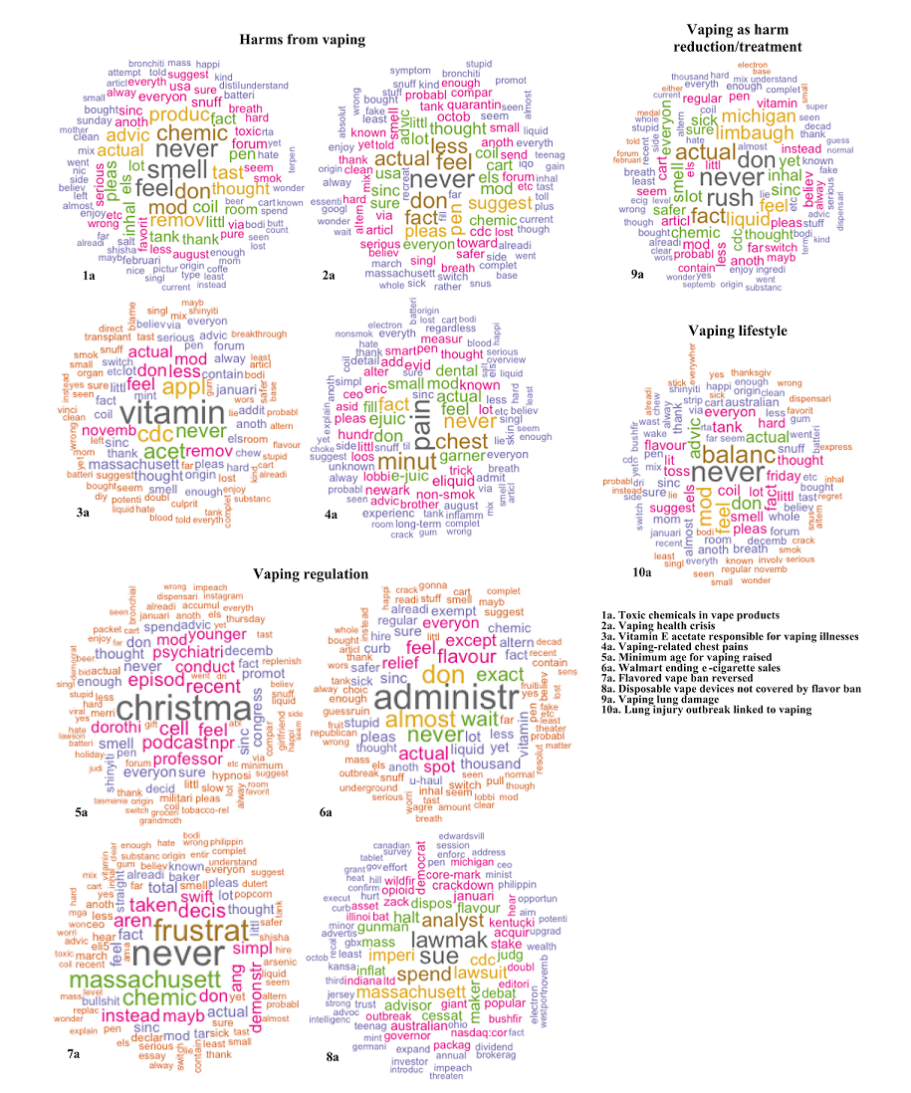
Major archetypes of vaping-related topics with example topics per archetype obtained by structural topic modeling for all observations (August 1, 2019, to April 21, 2020). The word clouds are generated from the weights of the top 100 terms within a topic. Terms with larger weights are depicted in larger font sizes. Terms with approximately the same weight are depicted in the same color.

**Figure 3:**
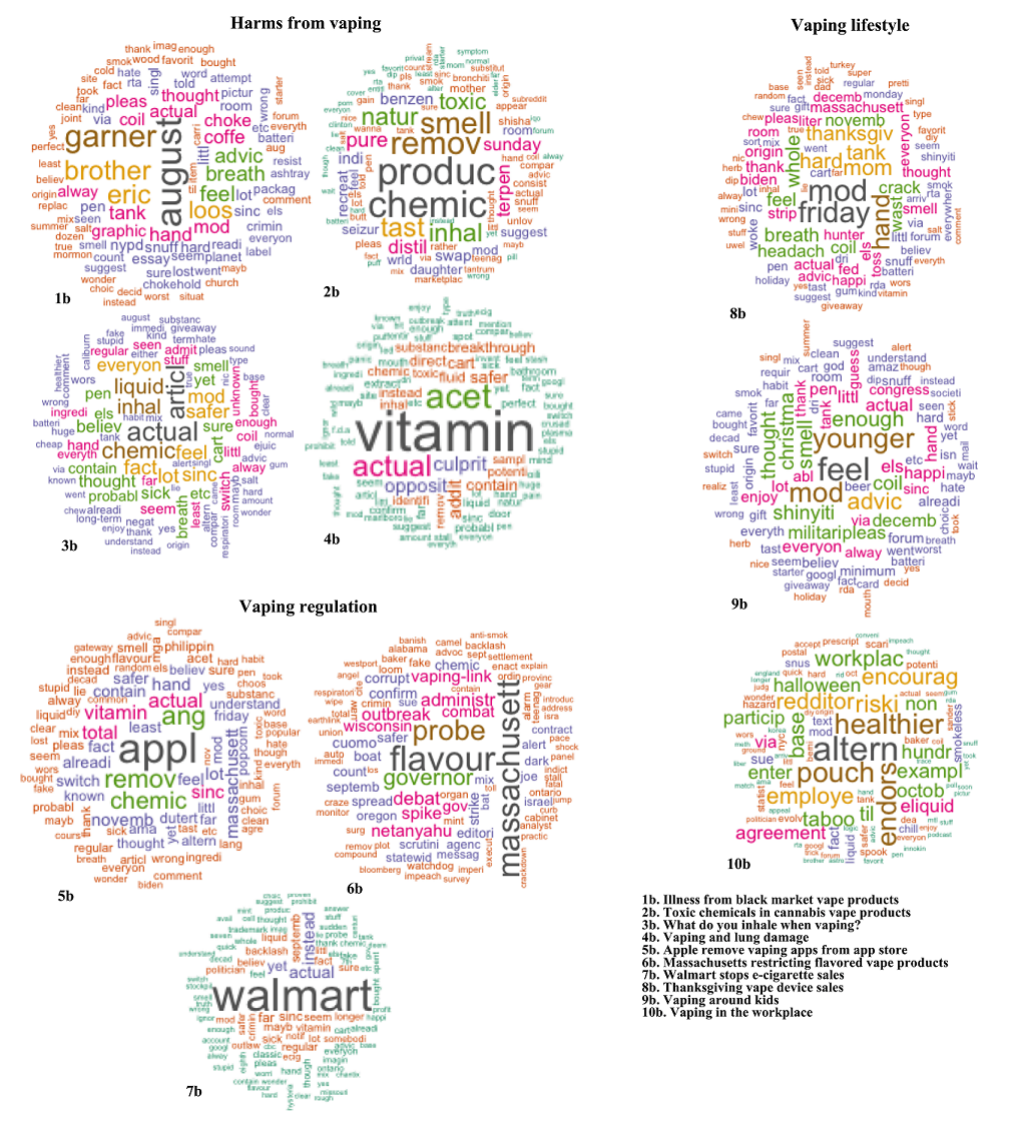
Major archetypes of vaping-related topics with example topics per archetype obtained by structural topic modeling before COVID-19 was reported to the WHO (August 1 to December 31, 2019). The word clouds are generated from the weights of the top 100 terms within a topic. Terms with larger weights are depicted in larger font sizes. Terms with approximately the same weight are depicted in the same color.

**Figure 4:**
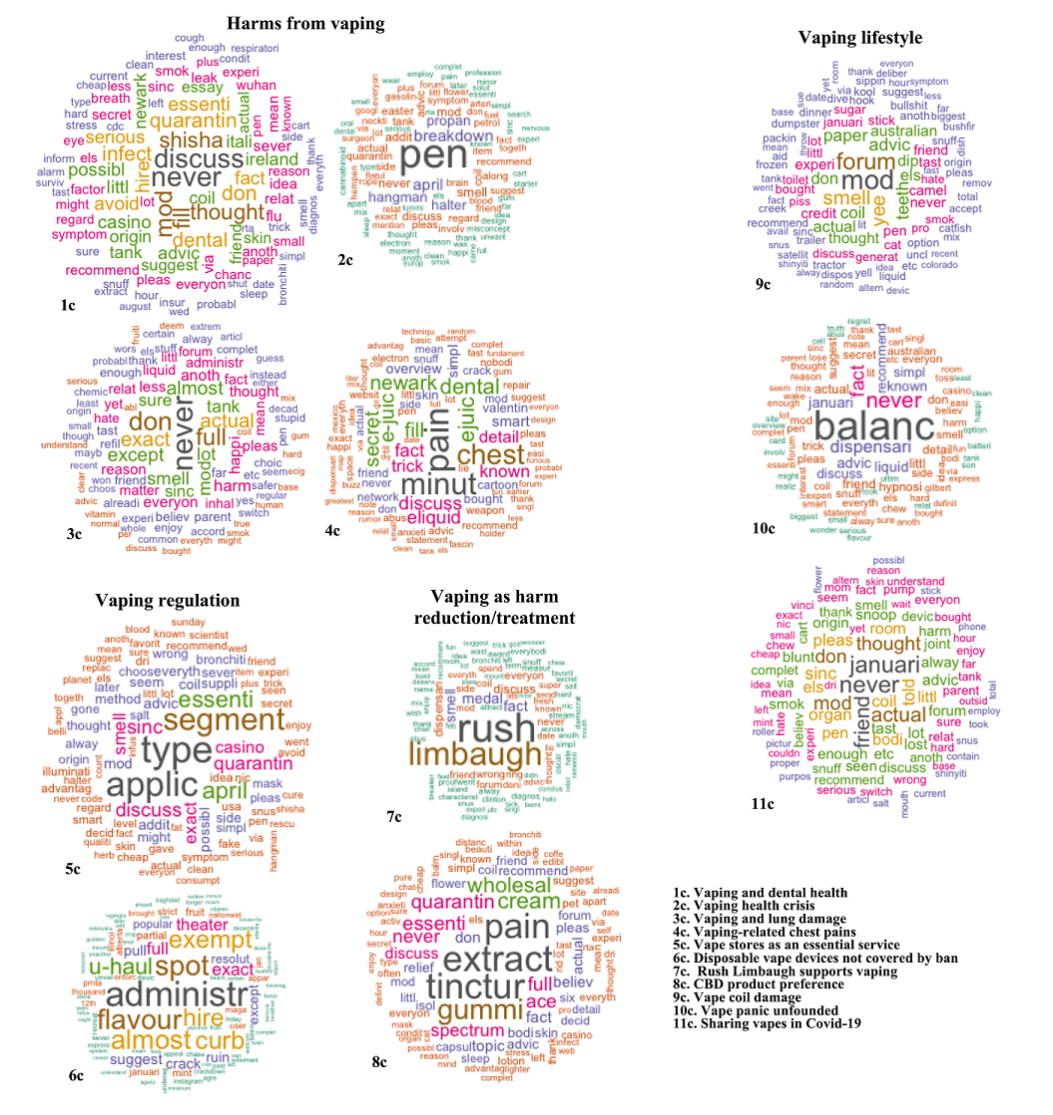
Major archetypes of vaping-related topics with example topics per archetype obtained by structural topic modeling after COVID-19 was reported to the WHO (January 1 to April 21, 2020). The word clouds are generated from the weights of the top 100 terms within a topic. Terms with larger weights are depicted in larger font sizes. Terms with approximately the same weight are depicted in the same color.

Figure 2 (all observations), Figure 3 (before COVID-19 was reported to the WHO), and Figure 4 (after COVID-19 was reported to the WHO) detailed results of the topic modeling analysis. Topics not directly relevant to our analysis were not indicated in the figures, such as the Australian bushfires and COVID-19 vaccine development. As detailed in the Methods section, we set the number of topics (k) as follows: whole data set (k=15); data prior to the report of COVID-19 to the WHO (k=20); and data after COVID-19 was reported to the WHO (k=20). We found that the models tended to reflect the same thematic structures of topics and differed only in granularity or level of detail.

Topic modeling captured significant events in the vaping environment, such as the vaping health crisis and Walmart stopping e-cigarette sales. The vaping health crisis referred to the 2019 emergence of vaping-associated pulmonary injury (VAPI) in the United States [39]. Walmart stopping e-cigarette sales denoted the September 2019 termination of vape sales at Walmart after the advent of VAPI [40]. These were likely significant events in the vaping narrative, as they emphasized the possible harms of vaping and were highly prominent in US media. Accordingly, these events were identified as individual topics. We organized the topics into the following groups or archetypes: Harms from Vaping; Vaping Regulation; Vaping as Harm Reduction or Treatment; and Vaping Lifestyle. We generated word clouds from the weights of the top 100 terms within each topic, and Figures 2–4 show sample word clouds for each of the three archetypes. Broadly, across the whole time period (Figure 2), the web-based vaping narrative was centered around harms from vaping and vaping regulation. Archetypes pre-COVID-19 (Figure 3) versus during COVID-19 (Figure 4) were largely similar, except that three archetypes were identified pre–COVID-19 (Harms From Vaping; Vaping Regulation; Vaping Lifestyle) but four archetypes were identified post–COVID-19 (Harms From Vaping; Vaping Regulation; Vaping as Harm Reduction or Treatment; Vaping Lifestyle). This suggests that the emergence of COVID-19 is related to the appearance of topics around vaping as a form of harm reduction or treatment. There was also variation in the topics within an archetype. For example, pre–COVID-19, the Harms From Vaping archetype included topics such as “illness from black market vape products” and “toxic chemicals in cannabis vape products.” After COVID-19 was reported to the WHO, example topics in the same archetype were “vaping and dental health” and “vaping and lung damage.” This suggests that while underlying themes in the vaping narrative are largely stagnant, specific topics in an archetype may vary over time. In line with the difference in archetypes before and during the COVID-19 pandemic, there were also different event-based topics before and during COVID-19. Before COVID-19, several topics represented events significant to vapers in that time period, such as “Walmart stops e-cigarette sales” and “Thanksgiving vape device sales.” These event-related topics were not present after COVID-19 was reported to the WHO. Instead, we noticed new event-based topics, such as “Rush Limbaugh supports vaping” and “disposable vape devices not covered by ban.” After COVID-19 was reported to the WHO, we noticed the emergence of topics specific to COVID-19 that were not present pre–COVID-19. These topics were “vape stores as essential service” (vape stores not being designated as essential services when COVID-19 lockdowns occurred in the United States) and “sharing vapes in COVID-19” (vape devices being possible sites of SARS-CoV-2 transmission). Similarly, a “CBD product preference” topic emerged after the COVID-19 report (Figure 4, word cloud 8c) under the Vaping as Harm Reduction or Treatment archetype that may be related to the advent of the pandemic. As indicated in the Methods section, we removed words around cannabis to provide for more nuanced analysis; accordingly, CBD and other related terms did not appear in the topic-based word clouds. This CBD topic was not present pre–COVID-19. The appearance of the “CBD product preference” topic may be related to vapers using CBD as a treatment for COVID-19. As a validity check, we examined the top 50 associated documents for the “CBD product preference” topic to validate the substantive meaning of the topic. Convenience sampling was not used to sample the top 50 topics; instead, we used the theta values of the topics, as detailed earlier. The number of top-ranked documents to be sampled was based on the methodology outlined earlier. There were 114,622 documents in total for the “CBD product preference” topic. These documents were read by two of the authors to determine validity. A third author resolved disagreements where necessary. As indicated above, interrater reliability was not used. We found that a majority of these top 50 documents (31, 62%) detailed CBD administered through vaping as a possible COVID-19 cure or protective agent. Example text fragments regarding how vaping CBD can prevent or treat COVID-19:

Pot smoke is the best expectorant I’ve ever used and fresh cbd oil or weed brownies are verrrrrrry healing, promotes good sleep and good healing. If you are a non-smoker, tobacco smoke should help you clear out your lungs if you have nothing else.

March 10, 2020

COVID-19 deaths invariably involve a ”cytokine storm,” an excessive, un-checked immune system response. Cannabinoids from cannabis, cbd in particular, can lower cytokine production naturally. research needed asap!

March 25, 2020

Example text fragments regarding websites marketing CBD for COVID-19 prevention or treatment:

Why hemp cbd flowers and a vaporizer are the best COVID-19 coronavirus prepping tools.

February 18, 2020

REDACTED COMPANY NAME applauds the use of cbd during the coronavirus outbreak

March 30, 2020

Toronto-based cannabis seller testing cbd’s effectiveness on reducing symptoms of coronavirus.

April 1, 2020

## Discussion

### Principal Findings

Our main finding was the emergence of discourse around vape-administered CBD treatment for COVID-19 when comparing web-based vaping narratives before and after the outbreak of COVID-19. Recent work suggested that CBD use may increase COVID-19 risks [41]. Other studies indicated that CBD may aid COVID-19 treatment outcomes [12,42]. Vaping CBD products as a treatment for COVID-19 is still largely unsubstantiated. Beliefs around CBD as a COVID-19 treatment, bolstered by marketing campaigns and early-stage research [11,12,42], may be responsible for the emergence of discussion around CBD.

There is limited empirical research on the intersection of the vaping narrative and COVID-19, especially around the emergence of CBD-related COVID-19 treatments and comparing the narratives before and during the COVID-19 pandemic. The role of web-based narratives in tobacco control, especially within social media, is a growing field of study [43]. We expand on past work that used novel computational approaches to examine trends in digital media to understand how these web-based behaviors may influence health behaviors [44,45]; our results indicate that the web-based environment is key to comprehending vaping and related health outcomes, especially in response to public health events. Previous research suggested the need to monitor social media content around tobacco to protect youth and mitigate tobacco use [43]. We expand on these studies, bolstering the need to surveil web-based tobacco content given our findings around increased discussion of inaccurate COVID-19 vape-administered treatments that are not evidence-based and may worsen health outcomes. A recent review detailed the role of misinformation in public health outcomes [17], and we expand on past work by providing evidence of how large-scale events may create misinformation in the health sphere. The strength of this study is our use of innovative computational methods to explore the content of the vaping narrative and how it is affected by COVID-19, comparing narrative content before the COVID-19 outbreak and after the pandemic took shape. This outcome measurement is key to understanding how vapers respond to COVID-19, enabling optimized treatment of vapers who develop COVID-19, and possibly minimizing instances of inaccurate information. Our findings around the use of CBD as a non–evidence-based and possibly injurious COVID-19 treatment, likely administered through vaping, are indicative of the earlier discussed point. Inaccurate information on the internet may create complications in COVID-19 treatment. Vapers who develop COVID-19 may use vape-administered CBD treatments; meanwhile, CBD is associated with reduced immune system functioning [41] and may heighten the disease progression of COVID-19. It is possible that upon contracting COVID-19, people may use vape devices to administer medication to themselves. Given the possibility of device malfunction [46,47], some individuals may further harm their health if they develop COVID-19 symptoms. As levels of misinformation around devices as a means to administer purported COVID-19 treatments increases, more people may share these modified devices; this creates possible sites of transmission [9,48] and may further increase SARS-CoV-2 transmission rates.

If health care professionals are aware that vapers with COVID-19 may use CBD as a treatment based on inaccurate information, these professionals may be better able to respond to vapers with COVID-19 who demonstrate CBD-related complications. Professionals can provide accurate information regarding COVID-19 to vapers who seek health care. Misinformation can also be combated with trusted information. Public health authorities can include COVID-19–specific information in targeted vaping-related messaging, perhaps mitigating consumption of inaccurate web-based information. Information-based campaigns can target inaccurate information in line with the topics identified by our results, such as vaping CBD as a COVID-19 treatment. There are several experimental interventions around reducing levels of inaccurate information [49,50], with some centering specifically on COVID-19 [51] and other health issues [17]. Interventions that harness similar techniques, such as asking respondents to judge information accuracy, may nudge individuals toward obtaining accurate information around COVID-19 and vaping. Interventions can also center on vaping-related health literacy in various media outlets, which may reduce misinformation about the topic [52]. Thus, our results may improve COVID-19 treatment for individuals who may have received inaccurate information around COVID-19 and vaping; they may also provide insight on reducing levels of misinformation among vapers during the pandemic.

Our findings have several implications. From a policy standpoint, we suggest that vaping forums be mandated to provide accurate data around the interactions between vaping and COVID-19. These efforts may reduce levels of inaccurate information around COVID-19 and vaping and may minimize any COVID-19 complications associated with vaping. Future research can identify changes in the vaping narrative as the pandemic progresses further, allowing public health authorities to adjust treatment provision for vapers at risk of contracting COVID-19. Future work can also address how inaccurate information on the internet can be mitigated, especially as the pandemic progresses.

### Limitations

Our results depended on the validity of the data collected through our textual query. We searched a wide range of web-based media and identified key themes that validated our results (eg, Walmart stops e-cigarette sales, Vitamin E acetate and vaping illness), and we are thus confident in the comprehensiveness of our data. We may have overlooked some slang terms for vaping and thus underestimated the web-based narrative. We did not obtain location data for individual text fragments; thus, we were not able to determine how COVID-19 cases in certain areas affected the narrative. Our data were drawn from August 1, 2019, to April 21, 2020, and we were not able to determine changes in the narrative after April. We were not able to collect all web-based vaping chatter and may have missed some themes. In future research, we will collect qualitative and survey data from vapers to enhance the current findings. We did not use interrater reliability for our qualitative analysis, and we will use such methods in future research.

### Conclusions

We demonstrated the advent of discourse around vape-administered CBD treatment for COVID-19 by comparing the web-based vaping narratives before and during the COVID-19 pandemic. The increase in CBD-related discussion within the vaping narrative may be due to the marketing of CBD products consumed through vaping as a COVID-19 treatment [11]. Our findings have implications for the management of COVID-19 among vapers and for monitoring of web-based content pertinent to tobacco.

## Appendix

Multimedia Appendix 1 List of sources from which data were obtained.

## Abbreviations

CBD: cannabidiol
e-cigarette: electronic cigarette
STM: structural topic modeling
VAPI: vaping-assisted pulmonary injury
WHO: World Health Organization
